# People with insomnia: experiences with sedative hypnotics and risk perception

**DOI:** 10.1111/hex.12388

**Published:** 2015-08-03

**Authors:** Janet M. Y. Cheung, Delwyn J. Bartlett, Carol L. Armour, Jason G. Ellis, Bandana Saini

**Affiliations:** ^1^ Faculty of PharmacyThe University of SydneySydneyNSWAustralia; ^2^ NHMRC Centres of Research Excellence: CIRUS (Centre for Integrated Research and Understanding of Sleep) and NeuroSleepThe Woolcock Institute of Medical ResearchSydneyNSWAustralia; ^3^ Clinical Management GroupThe Woolcock Institute of Medical ResearchSydneyNSWAustralia; ^4^ Sydney Local Health DistrictSydneyNSWAustralia; ^5^ Northumbria Centre for Sleep ResearchNorthumbria UniversityNewcastleUK

**Keywords:** benzodiazepines, health behaviour, hypnotics, insomnia, sedative antihistamine

## Abstract

**Background:**

Sedative hypnotics form an important part of managing insomnia and are recommended for short‐term use. It is standard practice for clinicians to inform the patient to use medications only ‘when required’, but the use of these medications is often chronic. Little is known about the impact of standard labelling/instructions on promoting appropriate medication use for managing insomnia.

**Objective:**

To explore patient medication‐taking beliefs, experiences and behavioural practices relating to the use of pharmacological/complementary sleep aids for insomnia.

**Setting and Participants:**

Specialist sleep/psychology clinics and the general community in Sydney, Australia.

**Method:**

Semi‐structured interviews were conducted with 51 people with insomnia using a schedule of questions to gauge their experiences, beliefs and current practices relating to insomnia medication use. Interviews were audio‐recorded, transcribed verbatim and subjected to Framework Analysis to identify emergent themes.

**Results:**

Participants held distinctive views about the safety and efficacy of complementary and pharmacological agents but do not intuitively turn to medications to resolve their sleep complaint. Medication use was affirmed through tangible medication‐taking cues due to the ambivalence in current instructions and labelling. Practices such as dosage modification, medication substitution and delaying medication use might be important drivers for psychological dependence.

**Conclusion:**

Current labelling and instructions do not necessarily promote the quality use of sedative hypnotics due to the variability in patient interpretations. Clarifying the timing, quantity and frequency of medication administration as well as insomnia symptom recognition would play a significant role in optimizing the role of pharmacotherapy in the management of insomnia.

## Introduction

Insomnia is the most prevalent sleep complaint in clinical practice. Psycho‐behavioural approaches are universally advocated as first‐line treatment across multidisciplinary best practice guidelines. Pharmacological treatment is effective, but recommended for short‐term relief only and should ideally be accompanied by behavioural treatments.^1^ However, sedative hypnotics (e.g. benzodiazepines, z‐drugs and sedating antihistamines) continue to be widely used for extended periods of time in the routine management of insomnia.^2^ Acutely, sedative hypnotics impair psychomotor vigilance which, depending on the time of administration, can extend into the next day and pose safety concerns around an individual's fitness to drive.^3^, ^4^, ^5^ Long‐term, extended sedative hypnotics use has been associated with cognitive impairment, falls, increased mortality, potential for dependence and abuse and the possible risk of developing dementia.^6^, ^7^ Although these effects are more pronounced in the elderly, this patient population remains the largest consumers of sedative hypnotics.^8^ Collectively, the adverse outcomes associated with sedative hypnotics contributes substantially, both directly and indirectly, to the health‐care expenditure.^9^ As a result, limiting patient exposure to pharmacological sleep aids has been an important agenda across different clinical contexts.^10^, ^11^, ^12^ Strategies include prescribing hypnotics on a ‘when required’ (PRN) basis, avoiding concomitant alcohol use, medication‐tapering programmes^11^, ^13^ and the development of low‐dose (i.e. 3.5 mg) ‘middle of the night’ sublingual formulations of zolpidem.^14^ Table 1 outlines how the respective strategies intend to promote the quality use of sedative hypnotics in clinical practice.

**Table 1:** Clinical interventions for promoting the quality use of sedative hypnotics in clinical practice

Strategy	Purpose
PRN prescribing	Promote intermittent use of medication to limit patient exposure to sedative hypnotics to only times of need
Avoid concomitant alcohol use	Prevent additive sedation when both sedative hypnotics and alcohol are consumed concurrently. Important safety implications for engaging in tasks requiring psychomotor vigilance the next morning (e.g. driving)
Medication‐tapering programmes	Limit exposure of these medications and curtail potential consequences (e.g. dependence, abuse, cognitive decline) associated with long‐term use
Low‐dose sublingual ‘middle of the night’ zolpidem formulation	Rapid onset of action and prevents residual sedation associated with taking standard doses of zolpidem (i.e. conventional tablet: 5–10 mg; controlled release: 6.25–12.5 mg) when experiencing sleep maintenance insomnia. Important safety implications for engaging in tasks requiring psychomotor vigilance the next morning (e.g. driving)

Whilst adverse consequences associated with the various hypnotics might stem from their inherent pharmacological properties, potential interactions arising from concomitant sleep aids use (e.g. OTC/CAMs), the extent to which the patient accurately interprets the labelling, instructions and precautionary measures also have important therapeutic, safety and health economic implications.^3^, ^15^ From the perspective of patient safety, risk communication (i.e. fitness to drive) is becoming an increasingly important area of focus in the provision of psychotropic medications including sedative hypnotics which are routinely used for the management of insomnia.^16^ Interventions include the use of dispensing software support tools^17^ and incorporating pictograms on ancillary warnings to enhance decision making around sedation risk.^16^ However, within the current scope of pharmacy practice in Australia, this message is only reinforced through counselling and basic labelling/ancillary warnings relating to concomitant alcohol consumption and sedation risk. Furthermore, despite the widespread use of complementary sleep aids, its connection between the perceived safety and prolonged use of pharmacological agents is unknown.^18^

As required ‘PRN’ self‐administration of medication has a long‐standing history in medical practice and is widely applied in disciplines ranging from pain management (e.g. opioids),^19^ gout management (e.g. colchicine in acute attacks)^20^ to insomnia (e.g. sedative hypnotics).^1^ The premise of doing so is to prevent the overuse of medications given the inevitable fluctuations in symptomology throughout the course of the respective disease states.^21^ However, the impact of this strategy on promoting the safe use of sedative hypnotics is unknown. In a study investigating patients’ health literacy, ‘take one pill by mouth every 12 h with a meal’ was only correctly interpreted by 53% (*n* = 359) of participants.^22^ It is expected that less explicit instructions such as ‘use when required’ which rely on patients’ self‐assessment could result in even higher rates of misinterpretation.^15^, ^22^, ^23^ Promoting intermittent sedative hypnotic use in the management of insomnia is also complicated by a combination of health system and psychosocial factors including the limited access to cognitive behavioural therapy for insomnia (CBT‐I), the patients’ unique set of treatment beliefs (i.e. perceived medication need/usefulness for addressing insomnia) and what is communicated to the patient by their health‐care provider.^24^, ^25^, ^26^ These factors are likely to dictate whether or not the patient chooses to take, how they take their sleep medications and whether or not these medications are being used safely.^27^ Therefore, the primary aim of this study was to explore patient beliefs and behavioural practices and their perception of sedation risk relating to the use of pharmacological and non‐pharmacological sleep aids for insomnia.

## Methods

### Recruitment

To capture diverse treatment experiences that are representative of the heterogeneous insomnia patient population, we invited clinicians from primary care practices (e.g. community pharmacy and general practice) and specialist clinics (i.e. Woolcock Institute of Medical Research Sleep Clinic and Headspace) to be involved in the study. Their primary role was to inform suitable participants about the nature of the study and the contact details of the researchers. In addition, advertisement flyers were displayed around these settings and the university community for participants to directly contact the researchers. Inclusion criteria for this study required participants to be ≥18 years of age, diagnosed with insomnia and/or referred to the study by their clinician or have self‐reported insomnia symptoms that meet the diagnostic criteria of the International Classification of Sleep Disorders 2nd Edition (ICSD‐2).^28^ People with insomnia were given a $50 voucher for their participation in the study. Study protocols were approved by the University of Sydney Human Research Ethics Committee (HREC: 2013/679). Informed consent and confidentiality agreements were sought from each participant prior to the interview.

### Data collection

Individual semi‐structured interviews were conducted by the first author (JC) either face‐to‐face or via telephone. The interview was guided by a schedule of questions (Appendix S1) exploring patients' perceptions towards medication use for insomnia, current medication‐taking routines and their understanding of standard labelling and instructions for pharmacological and/or complementary agents used to induce sleep. Interviews were digitally recorded then transcribed verbatim for analysis. Participants also completed a pre‐interview questionnaire which collected demographic information, a brief insomnia history, the Insomnia Severity Index (ISI)^29^ and Beliefs about Medicines Questionnaire General^1^ (BMQ‐Harm, BMQ‐Overuse).^30^, ^31^ Data collection continued until thematic saturation was independently achieved for the clinic (*n* = 22) and community (*n* = 29) population (i.e. Subsequent interviews did not provide additional thematic categories pertaining to the experiences of medication use of the respective patient groups).

### Analysis

Interviews were audio‐recorded, transcribed verbatim and de‐identified. Transcripts were analysed using Framework Analysis (FA) as described by Ritchie and Spencer and was carried out using QSR NVivo 10 software©.^32^ FA allows for the incorporation of multiple patient perspectives on the same phenomenon (i.e. medication experiences and practices). Analysis typically involves five key stages consisting of familiarization, identification of a thematic framework, indexing, chartering and finally mapping and interpretation. During *familiarization,* individual transcripts and field notes were read iteratively by JC and BS to understand some of the concepts and issues raised by participants. Emerging concepts were merged with the *a priori* concepts set out by the schedule of questions (see Appendix S1) and formed the basis of our preliminary *thematic framework*. New themes arising from subsequent interviews and analysis were discussed at research meetings and were iteratively incorporated into the developing thematic framework. The final thematic framework was applied systematically across the data set to *index* units of text corresponding to a particular theme, and earlier cases were also subject to re‐analysis. The indexed data were further re‐organized into thematic matrices containing thematic categories that were related to each other. In the final stage, mapping and interpretation, where cross‐case and within‐case associations were explored and discussed with the research team to conceptualize thematic categories that ultimately formed the set emergent themes presented.

## Results

### Sample characteristics

Fifty‐one semi‐structured interviews were conducted between August 2013 and May 2014 either through telephone (*n* = 36) or face‐to‐face (*n* = 15). The duration of interviews ranged from 39 to 139 min (median = 68 min). The 51 patients ranged between 19 and 69 years of age with a mean duration of insomnia of 10.2 and 11.2 years for the clinic (*n* = 22) and community population (*n* = 29), respectively. However, very little difference was observed across measures between the clinic and community patient sample. Table 2 summarizes participant demographic information. Patients reported using a broad range of sleep‐promoting agents including complementary, over‐the‐counter (OTC) and prescription items (Table 2). Complementary sleep aids included the use of proprietary formulations, herbal concoctions, teas and aromatherapy. Three participants (P8, P16 and P37) reported not having taken any sleep aids but having just explored (‘looked into’) different options.

**Table 2:** Participant characteristics

Variable	Community (*n* = 29) mean ± SD	Clinic (*n* = 22) mean ± SD
Male	9	6
Female	20	16
Age (years)	43.9 ± 13.4	42.3 ± 14.6
Mean duration of insomnia (years)	11.2 ± 10.6	10.2 ± 10.3
Mean ISI score	14.1 ± 5.0	12.3 ± 5.0
Mean BMQ (Harm) score	10.55 ± 2.5	9.2 ± 2.0
Mean BMQ (Overuse) score	11.8 ± 2.7	11.3 ± 3.3
Usage of sleep aids		
Nil	3	0
OTC only	2	1
CAM only	3	2
Rx only	1	1
OTC + CAM	5	2
Rx + CAM	9	4
OTC +CAM + Rx	6	12

BMQ, Beliefs about Medicines Questionnaire; CAM, Complementary and Alternative Medicine; ISI, Insomnia Severity Index; OTC, Over‐the‐counter medication; Rx, Prescription medication.

Synthesis of the qualitative data revealed three key themes: Treatment Beliefs, Taking Medication and Following Instructions. Where relevant participant extracts have been included to illustrate the organization of the emergent themes. Each participant is assigned a code with the letter ‘P’ followed by a number to indicate interview order and then either CL or CM to denote whether the participant was from a specialist clinic or community setting, respectively, followed by their gender and age.

### Theme 1: Treatment beliefs

#### The safety of medicines

People with insomnia across the two patient populations shared similar experiences and thoughts about the different sleep aids currently available. Irrespective of prior medication use, it was evident that participants had attached a different meaning and emotion to the different types of sleep aids currently available on the market. An important consideration among participants revolved around whether they were taking something that was ‘natural’ or a ‘chemical’. Despite knowing pharmaceuticals were more effective for inducing sleep people with insomnia disliked taking sleep medication and tended to favour ‘natural’ complementary products with the view that it is gentler on the body and has less potential to produce adverse effects and felt less concerned about taking them. Interestingly, prescription melatonin was also considered ‘natural’ and therefore ‘safe’.

I'll take something that isn't as effective so it doesn't have any negative consequences … I would generally lean towards the more natural solutions I guess. (P3_CM, Male, Age: 22)

Yeah ‘cause I feel like it's … with the herbal sure they're less intense but the flip side is you're less likely to get the bad side effects. (P9_CL, Female, Age: 22)

I felt it's a more natural remedy than the Temazepam. So I think I was less stressed about taking it… I was sort of bit more relaxed about taking the Melatonin … (P22_CL, Female, Age: 36)

When respondents were probed about the differences between OTC and prescription pharmaceuticals for managing their insomnia, it was generally agreed that prescription medications were more potent and more effective for promoting sleep. However, views towards the safety of the two were divided around respondents’ personal feelings towards medical supervision and the perceived strength of medication despite clear assignment of medication categories.^2^ Some participants rationalized that OTC sleep aids (e.g. doxylamine) was safer because they were readily accessible without prescription and therefore less potent and had a smaller potential for serious adverse outcomes compared to prescription sleep aids, whereas other participants believed prescription items were viewed to be safer on the premise that a doctor was medically supervising its use after careful history taking which may not be as extensive at the pharmacy during the provision of OTC sleep aids.

…the GP would prescribe it probably after a prolong conversation about, knowing what's going on with someone, they would prescribe what's required as opposed to just walking into a pharmacy and someone who may not fully understand in full what's going on and prescribes medication that could cause problems. (P48_CL, Male, Age: 36)

I don't know, because it's over the counter thing and I think it might be safer than the prescription, because prescription is probably stronger. (P51_CM, Female, Age: 56)

#### Function of the ‘sleeping pill’

People with insomnia saw the role of the pharmacological ‘sleeping pill’ purely as a means for resetting and regulating their sleep cycle with the expectation to cease medication once some sleep regularity had been restored. Interestingly, both treatment naïve (participant 8) and long‐standing users of sleep aids (participant 17) did not believe that pharmacological intervention would be suitable as a long‐term treatment solution as it does not address any of the underlying issues of insomnia. The perceived lack of a better alternative for managing insomnia had been the main reason for chronic medication use.

I think it (sleeping pill) will help to make my sleep cycle more regular and let me go to sleep at you know whatever time um… yeah I think it'd just make it more regular. That's the role I'd want it to have. (P8_CM, Female, Age: 27)

I think for short term or intermittent use, they're fine but long term no way. It's not good for long term because it's really addictive. (P12_CL, Female, Age: 29)

I take sleeping tablets, which I try not to‐, I am sort of trying to figure out the way to get off them, but pretty much obviously my body is addicted to them now, so I lost my natural ability to sleep. (P17_CM, Female, Age: 42)

On the other hand, complementary sleep aids (e.g. valerian and non‐prescription melatonin) served a number of roles for different people and reflect the heterogeneity of this patient population. People with insomnia elected to use these remedies prior to engaging with pharmacological medication. Even for those who regularly use pharmacological sleep aids, many still continue to use these remedies as a buffer to maintain a baseline level of sleep, to give the body a break from medication or were used as part of the troubleshooting process prior to taking pharmacological sleep aids.

Yeah, the Valerian. Yeah, I still take that actually, I still have one every night, which I‐, and I have chamomile tea too. So, they are both very natural things. (P11_CL, Female, Age: 61)

#### ‘Less is better’: attitudes towards pharmacological agents

Sleep was perceived as natural biological process and medication use violates the beliefs held by individuals and their contextual surroundings leading to their intrinsic dislike of taking medications for insomnia. Concerns stemmed from the fear of losing ‘natural sleep’, becoming addicted to medication and potentially harming the body with chemicals. People referred to ‘natural sleep’ in terms their perceived quality of sleep as well as the body's innate ability and need for sleep. Using medications to artificially induce sleep appears to be incongruent with the participants’ beliefs about the naturalness of sleep as a biological process and was further reaffirmed by their next‐morning perceptions of sleep quality. There was also fear that the body's natural sleep drive would diminish with continuous sleep medication use.

…even though I was just taking Temazepam just to get to sleep‐ but I was never happy about having to do so. (P22_CL, Female, Age: 36)

To be honest, I don't like the idea of the actual sleeping tablet – the reason being is because it sort of forces you to sleep as opposed to I guess um…making your mind relaxed and fall asleep naturally. (P36_CM, Male, Age: 21)

For many users of pharmacological sleep aids, taking as little as possible became a priority rather than following prescribers’ instructions or using symptoms to dictate their medication use and may stem from the aforementioned social expectations and sleep beliefs. Even when medication is taken, people with insomnia were often reluctant to take the full‐prescribed dose partly because of the potential consequence and partly to prevent medication losing its effect for when they really want to sleep. Respondents typically described taking half or a quarter of a tablet to ‘wait and see’ how the body responds. The remaining portions of medication were only taken if the initial dose failed to induce sleep and resulted in multiple subtherapeutic doses over the course of the night. However, similar behaviours were not reported in the use of complementary sleep aids.

I think probably then I was taking half as well. So as I said, less is better. But… yeah I think… do you know what I mean by that? I prefer to not take anything so I take as little as I possibly can. (P14_CL, Female, Age: 31)

I don't even know if I'd be happy to take something permanently to put me to sleep because I still believe, you know, sleep is a natural sort of thing that should happen and I know that I was always able to do that on my own without any drugs, so I would tend not to want to take a prescription tablet for sleeping, yeah. (P42_CM, Female, Age: 46)

### Theme 2: Taking medication

#### The meaning of ‘need’

When discussing how participants interpreted the standard instruction of ‘when required’ (PRN), many noted that the instruction is vague and that different people have different thresholds for ‘needing’ medication. While participants had been given the autonomy to decide when to take medication for their insomnia, they were also quite tentative when it came to exercising this autonomy and determining whether symptoms warranted pharmacological intervention. People with insomnia generally found the non‐specific nature of insomnia and the night‐to‐night variability in sleep patterns difficult for determining ‘when’ and ‘how much’ medication should be taken.

To me as required is… for me personally as required is when – when I can't sleep. If I can't sleep or if I think I'm not going to sleep that's when I should take it but it is kind of a vague term I suppose. (P12_CL, Female, Age: 29)

People with insomnia seem to struggle with trying to strike a fine balance between symptom control and not ‘taking too much’ to minimizing side‐effects. The effects of this ambiguity were more pronounced in those with co‐existing chronic conditions who frequently drew comparisons between the non‐specific nature of insomnia and the more prominent features of their other condition which they relied upon to administer medication. For example, participant 27 described relying upon the symptoms of ‘breathlessness and wheezing’ as a cue to use their asthma medication.

…it's a bit hard to determine when is required for the things like sleeping tablets, like what deems necessary compared to asthma medications when it is like ‘I can't breathe I need it’. (P27_CM, Female, Age: 19)

#### Justifying medication use

In response to insomnia, patients intuitively tried to induce sleep ‘naturally’ through using various passive sleep‐promoting strategies (e.g. drinking herbal tea, watching television, walking around the house or meditating). A key factor for medication use was dependent on the extent to which individuals perceived their insomnia symptoms to be severe enough to warrant pharmacological intervention. Severity thresholds were either based around night‐time routines or perceived next‐day demands. During the night, medication use was only considered appropriate after exhausting all possible strategies and/or spending a specific period of time sleeping and/or after a certain number of consecutive nights of poor sleep.

When I can't fall asleep and I know that I've tried the chamomile tea and the stretches or watching TV or reading and I think ‘now it's required’ I mean I've got 5 h to sleep, these are not gonna help or anything, I'm just going directly for Stilnox to get this sleep and make sure I can get good night sleep. (P45_CM, Female, Age: 51)

Alternatively, some people with insomnia relied on the perceived importance/demand of their next‐day schedule of activities to justify their medication use. They reasoned that they were willing to put up with symptoms of insomnia until they planned an important/demanding activity for the next day or the activity itself posed as a form of stressor that adversely affected their sleep – both of which warranted medication use.

Oh just if tomorrow's a really big experiment. I feel pressured that I need to do well, I'll take it. (P10_CL, Female, Age: 29)

#### Coping mechanisms

Participants seem to have developed a unique set of medication‐taking behaviours to allay their concerns around the use of pharmacological sleep aids. Part of this concern stems from the perceived harm of using medication as well as fear of becoming resistant to the sleep‐promoting effects of medication if used too frequently. This translated to a set of behaviours which involved periodically substituting pharmacological sleep aids for other medication classes (e.g. sedative analgesics) and/or rotating between complementary and medications as they were considered to be safer alternatives and therefore reducing the burden of the ‘harder’ drugs.

…I try and spread them around but, you know, taking the Blackmores, the herbal sleep things or Swisse sleep tablets, they basically do very little, if anything. (P35_CM, Male, Age: 64)

Well, sometimes I might take a couple of Avinols after dinner, hoping that I might relax then. If I'd had like 3 or 4 nights of taking a Stilnox, then I'd probably try the Periactin if I still was not going to sleep. (P49_CL, Female, Age: 52)

The dose of the pharmacological ‘sleeping pill’ was of great importance for people with insomnia and was often used to gauge the state of their sleep complaint. Taking less than the prescribed dose of medication was regarded as a positive health behaviour. Dose reduction was perceived as form of harm minimization for those who experienced difficulty coming to terms with their sleep medication use. People with insomnia also interpreted taking smaller doses as a sign of recovery and resuming some level of control over their sleep. To attest to these beliefs, participants often modified prescribed doses of medication of their own accord.

I take half, so I suppose that makes me feel that‐, because I'm taking half‐, that's okay. (P50_CM, Female, Age:52)

### Theme 3: Following instructions

#### Message from the health‐care professional

Interactions with health‐care professionals (HCPs) pertaining to the use of sleep aids were restricted to primary care doctors at the point of prescribing and/or community pharmacists when receiving an OTC product and/or a prescription item. The description of the nature and depth of the interaction was almost always dependent on the history of the participants’ medication use. For those encountering a HCP for the first time, the counselling interaction focused on restricting the time course of medications to ‘short‐term’, potential side‐effects and the addictive nature of hypnotics. Similarly, participants who sought OTC sleep aids from pharmacists recalled being counselled on the adverse effects of sedative antihistamines and the potential for first‐dose response sensitivity (i.e. residual sedation). With the exception of one participant (P11), complementary sleep aids rarely involved HCP input and the instructions on the packaging was relied upon. On the other hand, those with a long‐standing use of pharmacological sleep aids reported receiving relatively little counselling once their HCP confirmed that they had previously used the same medication. This subset of participants revealed much of what they understand about their medication was largely informed by their own experiences. However, neither patient group recalled being told the benefits of treatment or what to expect from treatment as part of their overall insomnia management plan.

…he would stipulate to try and not take it every night and um and I think I only got like sort of I think two or three scripts over you know over I suppose um a 5 month period um yeah he was really adamant that I didn't take them every night… (P1_CM, Female, Age 45)

…when he first gave it to me‐ ‘yep it's addictive um… don't take it any longer than um… a week or 2 weeks um…but he didn't really go through any information at the time. Um…it was only since I started taking it that I saw other people had bad reactions but since I've never had anything… any problems with it I don't worry about it too much. (P12_CL, Female, Age: 29)

Some chemists just might say ‘have you had this before’ I said ‘oh yeah I've had it before’, they say ‘ok here it is’. There's no specific warnings by‐, I assume there are warnings but they probably just say ‘have you had it before’… (P35_CM, Male, Age: 64)

#### Problems perceived, problems resolved

Both users and non‐users of pharmacological sleep aids agreed that ‘when required’ is an ambiguous instruction to follow because it was open for interpretation by the individual. Those with a long‐standing history of hypnotic use felt they had acquired extensive knowledge about their medication and believe that they had developed a sensible approach in their current medication‐taking routine in terms of gauging their symptoms and determining when and how much medication is needed. However, similar concerns resonated between both subsets of participants particularly with respect to the lack of an objective standard for determining symptom severity. People with insomnia related the instruction to their illness experiences and reasoned that everyone has a different threshold for tolerating symptoms, that is those with a low tolerance can potentially overuse medication, whereas those with a high tolerance may underuse medication. Similarly, when ‘prn’ is applied in the context of multiple wakenings, it can result in repeated medication use.

So if you can't sleep all the time and you're taking one every time you're trying to get to sleep, you could be trying to get to sleep two‐three times a day… maybe. (P23_CL, Female, Age: 42)

I suppose ‘when required’ it requires you to be able to identify when you are in need of the drug's assistance which is a‐, it's a bit risky actually, because it requires the person to be able to identify the symptoms and the extent of the symptoms. (P41_CL, Female, Age: 20)

Comments were made relating to the need for a more specific set of instructions to address current ambiguity. Suggestions made revolved around improving symptom recognition and setting quantifiable boundaries for the amount and timing of medication use.

More information about how often to take it with an actual number so if you can quantify that rather than being open and vague I think is better. (P8_CM, Female, Age: 27)

It probably would help to be more specific rather than saying ‘as required’… If it is one a day and your allowed to safely take one a day, then I think that needs to be specific, you need to say, ‘you're able to take one a day’. (P42_CM, Female, Age: 46)

#### Precautionary measures

There was a general familiarity with the ‘Label 1^3^ ’ ancillary due to its widespread application across the medications they have previously used (sleep and non‐sleep related). The extent to which people with insomnia paid attention to this label appeared to be influenced by their familiarity with the medication in question and the presence of psychosocial responsibilities which can potentially be affected. Those who had used a medication on an on‐going basis tended to pay little attention to the label and became increasingly complacent over the years because they were familiar with their body's response to medication.

I was pretty sure I wasn't meant to be driving and I did you know just built‐ you know I just got used to them um… yeah so I've been arrogant, very arrogant about that stuff. (P2_CL, Female, Age: 54)

Conversely, participants without prior experiences with the pharmacological sleep aids or those who were engaged in activities involving children (e.g. driving children to school) paid closer much attention to the warnings and exercised greater caution. Interestingly, participants enlisted similar resources and people to delegate tasks to.

Yeah, that's in my mind and when I took the Temazepam, I had to say to my husband you need to get up to the kids if something happens ‘cause yeah.’ (P23_CL, Female, Age: 42)

I'd rather say ‘Guys I am taking a week off, I am on this thing I've got to take this prescription, so I cannot drive any vehicle or operate any equipment for the next week. (P37_CM, Male, Age: 48)

When asked about the interpretation of this ancillary label, many thought the warnings and the effects of medication were restricted to the period of their sleep with the assumption that the medication would clear out of the system overnight. Participants reasoned that sedation is a desired outcome of taking a pharmacological ‘sleeping pill’ and found the warning to be somewhat counterintuitive. Nonetheless, of those taking pharmacological sleep aids (*n* = 43), over half reported experiencing residual sedation and described the symptoms in terms of ‘grogginess’ or a ‘hangover’. However, individuals did not perceive these non‐specific symptoms to be severe enough to warrant alternative arrangements, felt they had effective coping strategies or had adequate procedures to gauge whether or not they were affected by medication.

…as long as I feel like I can string a sentence together and I'm not wonky or anything then I'll still drive. If I'm just a little bit drowsy I'll drive. (P12_CL, Female, Age: 29)

## Discussion and conclusion

### Discussion

To our knowledge, this is the first in‐depth qualitative study exploring medication use from a diverse spectrum of the help‐seeking insomnia patient population (i.e. specialist clinic and community patient population). Research in this area has to date focused on addressing issues of drug dependence, drug burden and implementing medication‐tapering programmes.^11^ Our findings extend beyond this and reveal patient beliefs and counter‐productive behavioural practices (e.g. dosage modification) which might perpetuate medication use for insomnia.

Despite widespread hypnotic use for managing insomnia,^2^ both users and non‐users of pharmacological sleep aids shared negative views towards pharmacotherapy and appears to be an intrinsic response even across other more ‘acceptable’ conditions like asthma.^33^ We also noted the shift in attitude towards complementary medicines where individuals were willing to trade‐off perceived efficacy for lower risk of potential side‐effects. Prioritizing safety over efficacy has also been observed in another study exploring the use of natural sleep aids in a community pharmacy patient population where similar prioritizing strategy adopted by patients and may be a fundamental part of patients’ risk management strategies.^18^, ^34^ Despite the popularity of complementary therapies and its high levels of intrinsic acceptability among patients with insomnia, the efficacy and potential adverse consequences of such products remain inconclusive.^35^ Using ineffective treatments not only delays the resolution of symptoms but potentially perpetuates the development of chronic insomnia which becomes considerably more difficult to treat.^36^

Users of pharmacological sleep aids appear to closely adhere to HCPs’ instructions to limit medication use to only ‘when required’. However, patient interpretations are complicated by night‐to‐night sleep variability, negative societal messages constructed around the pharmacological ‘sleeping pill’^37^ and HCPs’ counselling focus on adverse effects. This is also reflected in patient BMQ‐Harm scores (Table 1) which indicate a general perception of medications doing more harm than good. For the individual, their logic of ‘less is better’ seems consistent with these messages as many begin to scrutinize over the accuracy of interpreting ‘required’ leading to tentative medication use. Uncertainty leads individuals to self‐titrate doses of prescribed and/or OTC sleep aids, spreading out single doses across the night, delaying medication use or periodically substituting the pharmacological ‘sleeping pill’ with complementary agents and sedating analgesics (e.g. codeine). Such behavioural patterns are described by Fainzang^34^ as strategies of *reduction* and *rearrangement* and may form the basis how individuals exercise ‘pharmacovigilance’ in their own social context.^38^ Similar compensatory health beliefs are often reported in the tobacco cessation literature where one ‘unhealthy’ behaviour is deemed to be neutralized by another ‘volitional healthy behaviour’.^39^

While participants considered their current practices to be positive health behaviours that effectively ‘neutralize’ perceived risks and medication load, they may in fact be compromising medication effectiveness. Many of the patient‐reported modifications render formulations subtherapeutic (e.g. quartering control‐release formulations) and may merely be exerting a placebo effect which falsely reinforces such practices as ‘safer’.^40^ Furthermore, the sporadic use of the various complementary remedies and ‘safer’ drugs (e.g. paracetamol) puts the patient at greater risk of drug–drug and/or herb–drug interactions particularly when these practices are considered ‘safe’ and ‘sensible’ and therefore not communicated to the clinician.^18^ Similar observations of medication down‐titrating/substitution among insomnia patients have also been documented by Henry.^41^ This might be an important set of *safety behaviours* through which the patient comes to terms with their medication use by reassuming control over sleep and gauge their recovery.

The compulsion to justify medication need can be explained by participants’ ‘less is better’ logic. Many rely on tangible medication‐taking cues through assessing next‐day activities or undertaking an extensive list of passive sleep‐inducing strategies to ‘wait for sleep to come’. The latter may be counter‐productive given passive strategies are ineffective^42^ and repeated distress from exposure of unsuccessful strategies prior to medication taking inadvertently conditions the patient to associate medication with sleep.^43^ This might be one possible mechanism through which psychological dependence occurs as the patient transitions from an acute to a chronic hypnotic user despite many viewing the pharmacological ‘sleeping pill’ as a short‐term solution and disliking the medication,^44^ much like staying in bed longer perpetuates insomnia.^45^ Delaying medication use at night also poses safety concerns relating to residual sedating effects the following day and have been implicated in road‐traffic accidents.^4^, ^5^, ^46^ However, most participants felt ‘Label 1’ instructions applied to the immediate hours following medication use and/or were confident about managing this ‘grogginess’ when driving. Similar levels of ambivalence have been observed in the use of the ‘yellow/black’^4^ label in the Netherlands.^16^ Including ‘categories of impairment’ and pictograms in warning labels have been shown to improve risk communication to the patient and positively modulate risk behaviours and thus warrants further research on its feasibility in Australian primary care.^47^

Whilst this study captured diverse experiences from a community (55%) and clinic (45%) patient population, there are a number of limitations. Our participants represent a self‐selected group who might be more actively engaged in the management of their insomnia and thus do not necessarily reflect the views of those who are less interested in the topic. Throughout the interview, participants were often asked to retrospectively report their treatment history and thus issues of recall accuracy should be considered. For a small patient subgroup without prior exposure to sleep aids (P8, P16 and P37), their perception towards medication was constructed around a hypothetical scenario, but nonetheless provide important insight that is representative of those in the early phases of help‐seeking and consulting the prescriber for the first time. Further, the vast majority of interviews were conducted using the telephone (*n* = 36) and while body language and facial expressions cannot be assessed, the participants may have been more comfortable about revealing their current medication experiences given the anonymity of the situation.

### Conclusion

A mismatch exists between HCPs’ provision of instructions and labelling and how the patient understands, interprets and applies this knowledge. Current instructions and labelling do not necessarily promote the quality use of hypnotics and could be contributing to the costly consequences of incorrect hypnotic use.^9^, ^48^ Instruction ambiguity poses further challenges for the patient struggling to come to terms with their medication use along with the variability of night‐to‐night sleep patterns. Refining labelling and education where clinicians work with patients to identify patient‐specific cues (i.e. symptoms ‘when unable to sleep’), set days for medication use, specifying doses/dosage intervals (e.g. one tablet to be taken at once) and timeframe (e.g. 30 min before desired bedtime) are potential ways for optimizing pharmacotherapy and preventing downstream chronic hypnotic use.

## Conflict of interest

No conflict of interest declared.

## Supporting information


**Appendix S1.** Semi‐structured interview guide.

## Notes

1 The BMQ general is made up of two components a General Harm scale (five items, total scores range from 5 to 25) and a General Overuse scale (three items, total scores range from 3 to 15) assessing beliefs about pharmaceuticals as a class of treatment.

2 In Australia, human medication categories fall under a wider classification system known as: ‘The Poisons Standard’ which includes industrial chemicals (e.g. pesticides) and veterinary chemicals. Within such a scheme, pharmaceuticals are designated as an OTC (i.e. Schedule 2 and Schedule 3) or a Prescription item (i.e. Schedule 4 or Schedule 8). This system is based on the premise of balancing access to medicines and self‐management of health conditions while protecting consumers from harm, particularly vulnerable consumer groups such as children.

3 Label 1 is an ancillary label used in Australia which states: ‘This medicine may cause drowsiness and may increase the effects of alcohol. If affected, do not drive a motor vehicle or operate machinery’.

4 The yellow/black label used in the Netherlands states: ‘This medicine can impair your reaction time. (driving‐using machines –playing on the street) Be careful in combination with alcohol!’
