# Supplementary material for: People with insomnia: experiences with sedative hypnotics and risk perception
Source: Health Expect. 2015 Aug 3;19(4):935–47. doi: 10.1111/hex.12388 (PMC5042066; doi:10.1111/hex.12388)
Supplement: Supplementary file 1 — Appendix S1. Semi‐structured interview guide. [file HEX-19-935-s001.docx]

| **Appendix A. Semi-structured interview guide** |
| --- |
| - Remembering from the last time you took a sleeping pill, how important are these medications for your sleep? - What role do these medications have in managing your insomnia? - How do find the instruction label that provides information about the use of your medications - Is information clear? - Any aspects you find confusing? - How do you understand the labeling and warnings provided by your doctor/ pharmacist? - PRN (When required? - Label 1 - How are you currently taking your sleep medications? Please describe your routine. - What can you still do the following morning after you take your sleep medication(s)? - What would you not do the following morning after taking your sleep medication(s)? |
